# Etiology and Treatment of Growth Delay in Noonan Syndrome

**DOI:** 10.3389/fendo.2021.691240

**Published:** 2021-06-04

**Authors:** Fernando Rodríguez, Ximena Gaete, Fernando Cassorla

**Affiliations:** ^1^ Institute of Maternal and Child Research, University of Chile, Santiago, Chile; ^2^ Pediatrics Department, Hospital Clínico San Borja – Arriarán, Santiago, Chile

**Keywords:** Noonan syndrome, growth delay, RAS/MAPK pathway, rhGH treatment, near adult height

## Abstract

Noonan syndrome is characterized by multiple phenotypic features, including growth retardation, which represents the main cause of consultation to the clinician. Longitudinal growth during childhood and adolescence depends on several factors, among them an intact somatotrophic axis, which is characterized by an adequate growth hormone (GH) secretion by the pituitary, subsequent binding to its receptor, proper function of the post-receptor signaling pathway for this hormone (JAK-STAT5b and RAS/MAPK), and ultimately by the production of its main effector, insulin like growth factor 1 (IGF-1). Several studies regarding the function of the somatotrophic axis in patients with Noonan syndrome and data from murine models, suggest that partial GH insensitivity at a post-receptor level, as well as possible derangements in the RAS/MAPK pathway, are the most likely causes for the growth failure in these patients. Treatment with recombinant human growth hormone (rhGH) has been used extensively to promote linear growth in these patients. Numerous treatment protocols have been employed so far, but the published studies are quite heterogeneous regarding patient selection, length of treatment, and dose of rhGH utilized, so the true benefit of GH therapy is somewhat difficult to establish. This review will discuss the possible etiologies for the growth delay, as well as the outcomes following rhGH treatment in patients with Noonan syndrome.

## Introduction

Noonan syndrome (NS - MIM# 163950) is a congenital disorder with an estimated incidence of one in 1,000 to 2,500 live births (1, 2). This estimate, however, may not be accurate since the condition may be underdiagnosed, particularly in mild cases, complicating the determination of its true frequency in the general population (3). The presence of familiar cases is consistent with an autosomal dominant inheritance pattern, but sporadic cases are more frequent (4). Although this syndrome presents three cardinal characteristics: distinctive facial features, postnatal short stature and cardiac anomalies (5), it coexists with minor signs such as cryptorchidism, delayed puberty, bleeding disorders, thoracic abnormalities, skin disorders, variable degrees of neurocognitive delay and predisposition to myeloproliferative disorders.

Noonan syndrome overlaps phenotypically with a number of related syndromes such as Neurofibromatosis type 1 (MIM#162200 - 1:3,000), Noonan syndrome with multiple lentigines (MIM #151100 - 1:100,000), Cardiofaciocutaneous syndrome (MIM #115150 - 1:200,000) and Costello syndrome (MIM #218040 - 1:400,000) (6). All of these conditions, including Noonan syndrome, are known as RASopathies, since their common molecular etiology are gain of function pathogenic variants of different components of the Ras/mitogen-activated protein kinase (MAPK) signaling pathway (7). This results in hyperactivation of the pathway, and possible deregulation of various cellular processes such as proliferation, survival, differentiation, migration, and metabolism. Up to date, the spectrum of genes with germline pathogenic variants associated with Noonan syndrome exceeds 20 (*PTPN11*, *SOS1*, *SOS2*, *KRAS*, *NRAS*, *RIT1*, *RRAS*, *RASA1*, *RASA2*, *MRAS, RAF1*, *BRAF*, *MAP2K1*, *MAP3K8*, *SHOC2*, *PPP1CB*, *SPRY1*, *LZTR1*, *MYST4*, *A2ML1*, and *CBL*). The role of some of these variants, however, remains controversial for some genes, such as *A2ML1* (8), or require further studies for others, such as *MAP3K8*, *SPRY1 and MYST4* (9). Approximately 73% of NS cases, however, exhibit a pathogenic variant in a sub-set of genes (*PTPN11* 50%; *SOS1* 11%, *RAF1* 5%, *RIT1* 5%, *KRAS* 1.5%), which allows for some genotype-phenotype correlations (**Supplementary Table 1**).

## Growth Delay in Noonan Syndrome

One of the cardinal features of Noonan syndrome is proportional growth retardation, occurring in most patients (10, 11), and represents the main cause of consultation to the clinician. These patients tend to have a birth length which is normal or slightly subnormal for gestational age. Cessans and co-workers found, in a molecular characterized cohort of 386 NS patients, that those patients with *PTPN11* and *RAF1* pathogenic variants were shorter at birth (12). In contrast, Malaquias and co-workers did not find this relationship between genotype and birth length in a cohort of 127 NS patients (13). Interestingly, both studies reported a significantly higher frequency of prematurity in NS patients, which could be associated with polyhydramnios, which is frequently observed in NS patients (14). Concerning postnatal growth, it usually follow a channel that is below, but parallel to the third percentile during infancy and childhood (12, 13). Even though deregulation of the RAS/MAPK pathway may explain this scenario, other factors may be involved, such as feeding problems (15) or a history of cardiac surgery. Patients with NS exhibit slow bone maturation and delayed puberty, which is associated with a limited pubertal growth spurt, leading to progressive growth retardation (16). A genetic correlation has been proposed, with patients with *PTPN11*, *RAF1*, and *KRAS* pathogenic variants showing more severely impaired growth than those patients with *SOS1* variants (12, 13). This may be explained by different magnitudes of RAS/MAPK activation for each genotype, and/or by the involvement of other signaling pathways related to RAS/MAPK (e.g. PI3K/AKT, JAK2/STATb5). Finally, adult heights, in different populations, have been reported below the third percentile in most affected patients (4, 17, 18). The average adult height for men with Noonan syndrome is 162.5 cm, and for women is 153 cm. It should be mentioned that women with Noonan syndrome reach their adult height by the end of their second decade of life, whereas men reach this milestone at the beginning of their third decade, suggesting that they undergo a limited but prolonged pubertal spurt.

### Pathophysiology of Short Stature

The pathophysiology of short stature in this condition remains poorly understood. Different potential mechanisms have been hypothesized, including growth hormone deficiency (GHD) (19–21), neurosecretory dysfunction (22) or mild GH resistance (23). The somatotrophic axis has been studied extensively in these patients, showing normal to elevated serum GH, with adequate responses to GH stimulation tests, but in some cases, they exhibit low mean GH concentrations during a 12-hour overnight profile. In addition, these patients show serum IGF-1 and ALS concentrations at the lower limit of the normal range, with IGFBP-3 concentrations usually within the normal range (20, 24, 25). The IGF-1 generation test, as reported by Bertelloni and co-workers in a well-defined pre-pubertal NS cohort with *PTPN11* pathogenic variants, showed an impaired responsiveness to GH stimulation compared with patients with short stature and healthy children (26). This hormonal profile suggests that some of these patients may show evidence of partial GH insensitivity, possibly at a post-receptor level.

### Growth Regulation in Murine Models

Several NS murine models have been developed (27). These models express pathogenic variants of *Ptpn11* (D61G) (28–30), *Sos1* (E846K) (31), *Kras* (V14I) (32), *Rit1* (A57G) (33) and *Raf1* (L613V) (34), in all tissues. All of them exhibit a normal birth length, but show reduced body length by weaning, which is concordant with the postnatal growth retardation observed in human NS patients. Regulation of growth has been investigated only in the murine model that expresses the pathogenic variants *Ptpn11* D61G/+ (28–30). De Rocca and co-workers (24) showed that serum IGF-1 levels are significantly lower in NS mice until the sixth week of life, when serum IGF-1 reach normal levels. A similar pattern was observed for serum IGF binding protein-3 (IGF-BP3), so the authors suggested that this scenario might be restricted to the growing period.

At a molecular level, Araki and co-workers (28) showed increased immunostaining of phospho-Erk (effector of RAS/MAPK pathway) at the developing limb buds of this model. In mouse embryonic fibroblasts, however, there was no difference in Erk activation in response to growth factors between heterozygous (D61G/+) and homozygous (D61G/D61G) mice. In addition, De Rocca (29) observed strong Erk phosphorylation in the liver of NS animals compared with their WT littermates, and Araki (28) suggested that Erk activation in this murine model might be dependent on the cell context. Furthermore, De Rocca demonstrated that inhibition of Erk phosphorylation significantly increased IGF-1 blood levels and body length in NS mice.

Tajan and co-workers (30) evaluated growth plate development in this NS murine model, and showed that the hypertrophic zone was smaller compared to WT animals. This finding correlated with a lower expression of transcripts associated with the hypertrophic stage, and with decreased alkaline phosphatase (ALP -chondrocyte differentiation marker) activity in NS chondrocytes. Moreover, increased basal and growth factor induced Erk1/2 phosphorylation was observed in NS hypertrophic chondrocytes. Interestingly, Tajan (30) showed that IGF-1 supplementation or inhibition of Erk phosphorylation partially corrects the growth retardation observed in NS mice. In addition, inhibition of Erk phosphorylation restored the length of the hypertrophic zones and ALP activity. Thus, Tajan hypothesizes that systemic IGF-1 deficiency is not the only mechanism responsible for poor growth plate development in NS.

In addition, De Rocca et al. proposed that autocrine/paracrine IGF-1 production may be altered in bone, and thus contribute to the impaired growth observed in NS. Several authors have suggested a direct participation (IGF-1 independent) of the RAS/MAPK pathway on physiological growth plate development (35, 36). Moreover, the important role of the RAS/MAPK pathway in growth plate development has been documented in achondroplasia and hypochondroplasia, the most common primary skeletal dysplasias in humans, which are caused by gain of function variants of the fibroblast growth factor receptor 3 (FGFR3) (37). It has been demonstrated that specific RAS/MAPK signaling inhibition in an achondroplasia murine model (Fgfr3 Y367C/+) leads to a significant recovery of bone growth (38).

Data from murine NS models provide evidence that the growth retardation in NS, at least in those cases associated with a specific *Ptpn11* variant, may be due either to partial GH insensitivity at a post-receptor level, and/or to an effect on RAS/MAPK activation. This murine model (*Ptpn11* D61G/+) not only reproduces several Noonan syndrome characteristics, but also allows for the understanding of the molecular process involved in the development of these features, in a tissue specific context. This has helped to clarify the effects of RAS/MAPK pathway deregulation in growth plate physiology. Further investigation of growth plate development, especially in those murine models with pathogenic variants associated with severe growth deficiency, as in the case of the murine model developed by Hernandez-Porras and co-workers (*Kras* +/V14I), would be very informative.

### IGF-1 Reduction and RAS/MAPK Hyperactivation

An intriguing aspect regarding linear growth in NS is the possible molecular relationship between GH induced hyperactivation of the RAS/MAPK pathway and reduced generation of IGF-1. The clues appear to reside in the interplay between this pathway and the JAK2–STAT5b signaling pathway, which induces IGF-1 production after GH receptor activation (39). Although the inhibitory effect of SHP2 which is activated in NS, on JAK-STAT5b signaling has been shown in some cell lines (40), the *Ptpn11* D61G/+ murine model does not reveal any change in STAT5b activity when stimulated with GH (29). Another issue to consider is the possible participation of the Src family of kinases (SFK), whose activation by GH is independent of the JAK2-STAT5b pathway, and results in induction of RAS/MAPK signaling (41, 42). Further research will be necessary to better understand the interplay between these intracellular pathways, and to clarify the mechanisms whereby pathogenic variants in RAS/MAPK, which prolong the intracellular signal induced by GH, results in a reduced generation of IGF-1.

## Growth Hormone Treatment for Noonan Syndrome

Treatment with recombinant human growth hormone (rhGH) has been shown to accelerate growth in patients with NS, but the benefit of long-term therapy over adult height is still the subject of some debate. The US Food and Drug Administration approved the treatment of NS patients with rhGH in 2007, and recently the European Medicine Agency (EMA) approved it too. In several other countries, such as Brazil, Israel, The Philippines, South Korea and Switzerland, rhGH is also licensed for this indication (43).

Most of the evidence regarding rhGH therapy in patients with NS originates from observational uncontrolled studies with relatively small numbers of subjects. Thus, very few controlled trials reporting adult height or near adult height in these patients have been published, but some longitudinal prospective trials and retrospective studies based on post-marketing studies are available. These data are difficult to compare, however, due to the heterogeneous treatment protocols employed, as well as the different cohort selection criteria, age at onset of therapy, rhGH dose employed, and duration of treatment. Some of the series published show a significant gain in adult height of approximately 1.4 ± 0.8 SDS (9.5 ± 5.4 cm) (44), but others have not confirmed these findings (45). The published studies can be classified into post-marketing observational studies, generally financed by the pharmaceutical industry, and prospective and retrospective clinical trials conducted in selected groups of patients.

### Short-Term Studies**

Several short-term studies with less than one year of rhGH therapy have reported a transient increase in height velocity and height SDS in NS patients, as described for other clinical conditions characterized by short stature (20, 46, 47). In addition, Şiklar and co-workers reported the effect of rhGH treatment during three years in a group of 47 patients with NS (48). In this study, the height standard deviation score (HSDS) increased from -3.62 ± 1.14 to -2.85 ± 0.96, and this increase was significantly greater when compared with the patients who did not receive rhGH (**Table 1**). Longer-term studies, such as those published by MacFarlane (49) and Lee (50), also reported an increase in height velocity and height SDS during rhGH therapy in patients with NS (**Table 1**). The MacFarlane study is the only controlled trial reporting data from 31 children (23 treated and 8 untreated), which showed that after 3 years of rhGH therapy, the treated group gained an average of 3.3 cm more than the untreated group (49). In addition, Lee compared the responses to rhGH therapy in patients with NS and Turner Syndrome (TS), which were enrolled in the Nordinet International Outcome Study (IOS), and demonstrated that both groups of patients achieved a similar response after 4 years of rhGH therapy (50). In NS and TS patients, the 4-year adjusted ΔHSDS were 1.14 ± 0.13 and 1.03 ± 0.04, respectively. Based on untreated, disease-specific references, the four year adjusted ΔHSDS for NS and TS were 1.48 ± 0.10 and 1.79 ± 0.04 (p<0.0001) respectively, with ΔHSDS being greater in younger patients at the onset of treatment. Finally, Horikawa and co-workers investigated the long-term efficacy and safety of Norditropin^®^ in a 4 year randomized, double blind, multicenter trial in prepubertal patients with NS (51) (**Table 1**). The patients treated were randomized 1:1 to receive rhGH at a dose of either 0.033 or 0.066 mg/kg/day. Height SDS increased from -3.24 to -2.39 in the low dose group (change in HSDS was 0.85), and from -3.25 to -1.41 in the high dose group (change in HSDS was 1.84) after 208 weeks of therapy (p<0.001). It should be noted that the authors did not observe any serious adverse events during therapy with rhGH.

**Table 1:** Published short-term studies in patients with Noonan syndrome treated with rhGH.

	Macfarlane et al. (49)	Lee et al. (50)	Siklar et al. (48)	Horikawa et al. (51)
Patients, n	23	30	47	51
Publication year	2001	2015	2016	2020
Patient population	United Kingdom	USA	Turkey	Japan
Duration of therapy (years)	3	4	3	4
Age at start, years (range or *SD*)	9.3 ± 2.6 (4.5 to 14.0)	8.39 ± 3.45 (2.4 to 14.3)	9.8 ± 3.4 (0.08 to 17.8)	6.06 (*2.25*)
Height at start, SDS (SD)	-2.7 ( ± 0.4)	−2.64 ( ± 0.96)	-3.62 ( ± 1.14)	-3.24 ( ± 0.6) [0.033] -3.25 ( ± 0.71) [0.066]
rhGH doses (mg/kg/day) (SD)	0.047	0.048 ( ± 0.011)	0.035	0.033/0.066
Height at 1 year, SDS (SD)	-2.2 ( ± 0.6)	ND	ND	-2.4 [0.033] -1.78 [0.066]
Height gain at 1 year, SDS (SD)	0.5	0,50 ( ± 0,03)	0.4 ( ± 0.44)	0.84 [0.033] 1.47 [0.066]
Growth Velocity at 1 year (cm/year)	8.4. ± 1.7	ND	ND	ND
Genetic analysis	ND	ND	*PTPN11* (n:39)	*PTPN11*, *SOS1*, *KRAS*, *RAF1*, *BRAF*, *SHOC2*, *RIT1*
Height at therapy end, SDS (SD)	-1.9 ( ± 0.9) (n:23)	-1.48 ( ± 0.09)	-2.85 ( ± 0.96)	-2.39 [0.033] -1.41 [0.066]
Height gain at therapy end, SDS (SD)	0.8	1.14 ( ± 0.13)	0.77 ( ± 0.42)	0.85 [0.033], 1.84 [0.066]

Data are expressed as means [Adapted from Carcavilla et al. (43)].

### Adult Height/Near Adult Height

Adult or near-adult height data have been reported in four studies, and there is additional information from four patients documented by Municchi and co-workers in 1995 (52). None of these studies included a control group, so the growth of the treated patients was compared with historical growth references for NS. Unfortunately, some of these references are from cross sectional studies without genetic confirmation of the condition, and with small numbers of patients. As observed in **Table 2**, age at start of treatment and duration of rhGH varied widely among these studies, whereas mean heights at the start of treatment were similar. These studies show a relatively large variation in the height gain observed during rhGH therapy (0.6 – 2.0 SDS), with the best results reported in patients who started treatment at a younger age.

**Table 2:** Published adult height data in patients with Noonan syndrome treated with rhGH.

	Kirk et al. (45)	Osio et al. (53)	Raaijmakers et al. (54)	Noordam et al. (55)	Romano et al. (16)	Ranke et al. (56)
Patients with adult height, n	10	18	24	29	65	140
Data source	KIGS UK	Random study	KIGS world	Random study	NCGS observational	KIGS observational
Publication year	2001	2005	2008	2008	2009	2019
Age at start, years (range or *SD*)	10.2 (8 to 15)	8.2 (3 to 14)	10.2	11.0 (6 to 18)	11.6 (*3.0*)	9.6 (4.6 to 14.6)
Height^1^ at start, SDS (range or *SD*)	-3.1 (-4 to -2)	-2.9 (-4 to -2)	-3.3	-2.8 (-4 to-2)	3.5 (*1.0*)	-3.3 boys; -3.7 girls
rhGH dose, mg/kg/day	0.035	0.033/0.066	0.035	0.05	0.05	0.037
Duration of therapy, years (range or *SD*)	5.3 (2 to 8)	7.5 (4 to 12)	7.6	6.4 (3 to 10)	5.6 (*2.6*)	> 3
Height gain^1^, SDS (range)	0.6 (-0.2 to -1)	1.7 (0.4 to 0.3)	0.97	1.3 (-0.6 to 2.4)	1.2 boys; 1.5 girls	1.2 boys; 1.3 girls
Height gain^2^, SDS (range)	0.8	1.7 (0.5 to 3.1)	0.61	1.3 (-0.2 to 2.7)	0.7 boys; 0.3 girls	ND

Data are expressed as means; ^1^according to Tanner; ^2^according to Noonan reference [Adapted from Dahlgren (57) and Cascavilla et al. (43)].

More recent data suggest that an additional spontaneous height gain of 1.0 SDS may occur during the end of the second decade of life in girls with NS, with a further gain of 0.57 SDS at the start of the third decade in boys with NS (58). Therefore, the potential positive effects of a late pubertal growth spurt should be considered in the projections of adult height in patients with NS. Osio and co-workers reported adult height in 18 children with NS who were treated with rhGH during a mean period of 7.5 years (53). These authors reported an increase in adult height from −2.9 to −1.2 SDS after completion of rhGH treatment in this uncontrolled study (**Table 2**). In addition, they observed a further height gain of 0.9 SDS in males and 0.5 SDS in females during late puberty, so these patients attained their adult height at a mean age of 19.5 years (range 17–21 years).

Noordam and co-workers have also published adult height data in 29 patients with NS (55). The mean adult height SDS of these patients was –1.5 and 1.2 according to National and Noonan standards, respectively. The mean adult height for boys was 171 cm and 157 cm for girls indicating a final height gain of 1.3 SDS, which corresponds to approximately 9.5 cm (**Table 2**). Linear regression analysis showed that age at start of puberty made the only statistically significant contribution to the gain in adult height in this series. In addition, Tamburrino and co-workers reported growth data in patients affected by RASopathies with a molecularly confirmed diagnosis. Adult height was reported in 33 patients, including 16 patients with GH deficiency (GHD). This study showed that long-term rhGH therapy accelerates growth in GHD subjects affected by RASopathies, normalizing adult height for Ranke standards, although most patients did not show the characteristic catch-up growth observed in patients with isolated GHD treated with rhGH (59). Recently Malaquias and co-workers, reported 42 patients (35 *PTPN11*+) who were treated with rhGH, and followed 17 until adult height. This study showed that *PTPN11*+ patients had a greater growth response than *PTPN11*– patients. Among the patients that reached adult height, AH-CDC SDS and AH-NS SDS were –2.1 ± 0.7 and 0.7 ± 0.8, respectively. The total increase in height SDS was 1.3 ± 0.7 and 1.5 ± 0.6 for normal and NS standards, respectively (60).

### Observational Post-Marketing Studies

The KIGS program (sponsored by Pfizer) for NS patients from the United Kingdom, showed a mean increase in adult height of 0.8 SDS. This was a cohort of 10 very short patients (-3.0 SDS) treated for an average of 5.3 years, whose age at initiation of therapy was relatively late (20). A study by Raaijmakers and co-workers, reported near adult height in 24 patients after at least 4 years of treatment. The median age at the start of treatment was 10.2 years and the median duration of rhGH treatment was 7.6 years. Median gain in height was 0.61 SDS according to Tanner standards, and 0.97 SDS according to Noonan standards (54) (**Table 2**). In addition, Romano and co-workers, evaluated the response to rhGH therapy in 252 patients by analyzing growth data from children with NS who were enrolled in the National Cooperative Growth Study (sponsored by Genentech). These authors reported near adult height in 65 patients, and the mean height gain was 1.4 SDS, which represents a mean gain of 8.9 cm for girls and 10 cm for boys (16) (**Table 2**). These results were compared with children treated with rhGH for idiopathic growth hormone deficiency and Turner Syndrome. Children with NS monitored for at least 4 years had a significant increase in height SDS scores, and their height velocity was greater than in girls with Turner syndrome treated for the same period. The most recent study of the KIGS program, published in 2019, describes a cohort of 140 patients treated up to adult height (**Table 2**). The males reached an adult height of 163.7 cm (-2.0 SDS), and the females 149.5 cm (-2.5 SDS), with an increase in 1.1 SDS for boys and 1.3 SDS for girls, after a mean duration of treatment of 6.8 and 6.3 years, respectively (56).

### Variables Correlated With Increased Growth

Several authors (50, 61) have shown that early initiation and longer duration of therapy are associated with a greater height gain. Thus, initiation of rhGH therapy at a relatively late age may explain the modest results observed by some authors (20). In addition, the duration of rhGh therapy before puberty and the height SDS at the onset of puberty are important contributors to near adult height in patients with NS. This suggests that growth optimization may be possible with earlier initiation and longer duration of rhGH therapy in patients with this condition (56, 59). No significant correlation with rhGH dose nor with gender, however, has been observed in most of these studies (56, 61). In addition, whether the clinical phenotype is moderate or severe does not appear to be a predictor of the response to rhGH therapy. Both phenotypes respond similarly to rhGH treatment despite the significantly higher mean GH levels observed in the severe phenotype (62). In some short-term studies, a correlation between the growth response and the genotype has been suggested, with a lower growth response in patients who carry a *PTPN11* pathogenic variant. This finding has not been confirmed by other authors, however, who have not observed any differences in height gain, height velocity SDS, adult height and/or serum IGF-1 in NS patients with or without *PTPN11* pathogenic variants (16, 56).

Serum IGF1 has been used to monitor the response to rhGH therapy in NS, and usually rises during rhGH therapy in parallel with an increase in height SD (61). Two studies have investigated the response to rhGH treatment in NS patients with GH deficiency. The study by Tamburrino and co-workers (59) showed that GH-deficient patients, treated with the doses of rhGH employed for classical GH deficiency, had a gain in height SDS that placed them within the normal range for NS, but not for the normal population. This suggests that GH deficiency is not the only explanation for the short stature observed in some of these patients, and that the doses of rhGH employed for patients with classical GH deficiency are insufficient to normalize height in NS.

Zavras and co-workers (63) assessed growth response following GH therapy (0.25 mg/Kg/week) in five GH-deficient NS (NSGHD) patients and in five idiopathic GH deficient patients (IGHD). At the beginning of GH treatment, height velocity were statistically lower in NSGHD children than in IGHD. During the first three years of rhGH therapy, the NSGHD patients showed a slight increase in height (from −2.71 SDS to −2.44 SDS) and in height velocity (from −2.42 SDS to −0.23 SDS), but these parameters remained significantly lower than in IGHD children. In addition, after five years of rhGH therapy, height gain was higher in IGHD children (mean 28.3 cm) than in NSGHD patients (mean 23.6 cm).

### Safety of rhGH Therapy

The overall experience with rhGH therapy in most short children is relatively reassuring. However, some studies have shown an increased risk for cardiovascular events and second tumors in children with a primary tumor treated with rhGH during childhood and adolescence (64, 65). Other publications, however, have not shown a significant increase in overall mortality in low-risk patients, such as those with isolated GH deficiency or idiopathic short stature. In patients with an inherent increased mortality risk, such as those with a predisposition or harboring tumors, the increased mortality rates appear to be related the underlying diagnosis rather than to rhGH therapy (66).

We should state, however, that none of the published series with patients with NS has reported serious adverse effects during rhGH therapy. Key parameters, such as BMI and markers of carbohydrate metabolism usually remain within the normal range during rhGH therapy (47). Two prospective studies specifically designed to evaluate cardiac anatomy and function after 1 and 4 years of rhGH therapy, did not show any change in myocardial function, or of ventricular wall thickness (20, 67).

In addition, RAS/MAPK hyperactivation is a common feature of many types of somatic malignancies. NS represents a cancer-prone syndrome and is associated with increased risks for childhood leukemia and solid tumors, as shown by Kratz and co-workers (68). Some genotypic variants of NS are associated with the development of neoplasms, such as the substitution of codons p.Asp61 and p.Thr73Ile at SHP2 (*PTPN11*), which are associated with Myeloid Leukemia (69). Even though there are a few cases described in the literature, most studies have not shown an increase in the incidence of neoplasms in patients with NS treated with rhGH. It should be stated, however, that there are no long-term studies specifically designed to address this outcome, so continuing surveillance is of paramount importance in NS patients.

## Conclusion

Despite the recent description of new genes associated with Noonan syndrome, and the availability of several murine models with pathogenic variants of the RAS/MAPK pathway, the molecular pathophysiology for the growth delay observed in these patients has not been elucidated. One of the most intriguing questions is the possible molecular link between RAS/MAPK hyperactivation and IGF-1 expression, and its relationship with growth plate development. In addition, therapy with rhGH increases height velocity in patients with NS, but firm conclusions regarding the effects of this therapy on adult height are not available. Therefore, there is a need for large controlled clinical trials in patients with this condition, in order to accurately assess the effects of rhGH therapy over adult height. In addition, given the complexity of this disorder, in terms of the high prevalence of cardiac defects and the possible risks of malignancy, it is very important to maintain a strict surveillance of these patients.

## Author Contributions

FR developed the Introduction and the Growth delay in Noonan syndrome sections. XG developed the Growth hormone treatment for Noonan syndrome section. FC developed the Conclusion section and perform sections integration and manuscript revision. All authors contributed to the article and approved the submitted version.

## Conflict of Interest

The authors declare that the research was conducted in the absence of any commercial or financial relationships that could be construed as a potential conflict of interest.
