# Supplementary material for: Etiology and Treatment of Growth Delay in Noonan Syndrome
Source: Front Endocrinol (Lausanne). 2021 Jun 4;12:691240. doi: 10.3389/fendo.2021.691240 (PMC8212989; doi:10.3389/fendo.2021.691240)
Supplement: Supplementary file 1 [file Table_1.docx]

| **Gen** | Chromosomal localization | Frequency of pathogenic variant in NS | Associated phenotype |
| --- | --- | --- | --- |
| ***PTPN11*** (MIM 176876) | 12q24.1 | 50% | Typical facies. Valvular pulmonary stenosis. Tendency to bruising. Short stature. Cryptorchidism. Familiar cases. |
| ***SOS1*** (MIM 182530) | 2p22.1 | 11% | Skin manifestations (follicular keratosis, scarce and/or curly hair, scarce eyebrows). Low frequency of short stature and intellectual disability |
| ***RAF1*** (MIM 164760) | 3p25.2 | 5% | Hypertrophic cardiomyopathy (sometimes neonatal). Pigmented macules |
| ***RIT1*** (MIM 609591) | 1q22 | 5% | Hypertrophic cardiomyopathy (sometimes neonatal). Juvenile myelomonocytic leukemia. Cryptorchidism. Low frequency of short stature, skin involvement and intellectual disability |
| ***KRAS*** (MIM 190070) | 12p12.1 | 1,5% | Cognitive impairment, skin alterations (follicular keratosis, scarce and/or curly hair, scarce eyebrows). Short stature. |
| *Mendelian Inheritance in Man* (MIM) *gene code*; [*Adapted from Carcavilla et al.* (70)] | | | |

**Supplementary Table 1:** Top five genes in Noonan syndrome and its associated clinical features.

70. Carcavilla A, Suárez-Ortega L, Rodrıǵ uez Sánchez A, Gonzalez-Casado I, Ramón-Krauel M, Labarta JI, et al. Noonan Syndrome: Genetic and Clinical Update and Treatment Options. Pediatr (Barc) (2020) 93:61.e1–61.e14. doi: 10.1016/j.anpedi.2020.04.008
